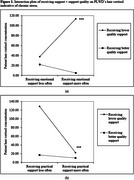# Support Quality Matters: Receiving Support and Hair Cortisol in People Living With Early‐Stage Alzheimer's Disease

**DOI:** 10.1002/alz70856_099117

**Published:** 2025-12-24

**Authors:** Meng Huo, Kyungmin Kim, Wen Wang

**Affiliations:** ^1^ UC Davis, Davis, CA, USA; ^2^ Department of Child Development and Family Studies, Seoul, Seoul, Korea, Republic of (South); ^3^ University of Oregon, Eugene, OR, USA

## Abstract

**Background:**

Receiving support typically creates stress in later life, but it is an inevitable routine for people living with Alzheimer's disease (PLWD) that disrupts their daily functioning. The current study examined the association between receiving support and chronic stress in this unique context, adopting hair cortisol concentrations as a non‐invasive, objective physiological marker of chronic stress in PLWD. Further, we explored whether the quality of support that PLWD received from their spousal caregivers moderated this association.

**Method:**

Participants included 62 older adults living with early‐stage AD (*M*_age_ = 77.44, 36 men). They self‐reported the frequency and quality of support received from their spousal caregivers. Hair samples were collected from the posterior vertex to assess cortisol concentrations. Three centimeters from the scalp end were assayed using enzyme immunoassay (EIA) method to reflect chronic stress over the past three months. Spousal caregivers reported their own and PLWD's demographic characteristics.

**Result:**

Receiving more frequent emotional support and less frequent practical support from spousal caregivers was associated with higher hair cortisol concentrations (indicating greater chronic stress) in PLWD. Yet, these associations were only evident when the quality of support was low (see Figure 1). When PLWD received high quality support from spousal caregivers, the frequency of receiving support was not associated with PLWD's chronic stress.

**Conclusion:**

This study extends the caregiving literature by incorporating an innovative, physiological measure of chronic stress and offering insights into PLWD's stress as they adapt to receiving intensive care from their spousal caregivers. When the quality of support is low, receiving emotional support more often elicits greater chronic stress in PLWD, whereas receiving practical support reduces stress. Findings highlight the importance of enhancing caregiving quality and shed light on the development of interventions that can guide caregivers in providing tailored, targeted support and reduce PLWD's chronic stress.